# Plasma GLP-1 (Glucagon-like Peptide-1) Depletion Is Correlated with Dysregulation of Adipocytokine in Type 2 Diabetic Patients With or Without Metabolic-Associated Fatty Liver Disease (MAFLD): A Cross-Sectional Study Related to Gender-Sex Disparities

**DOI:** 10.3390/ijms27031218

**Published:** 2026-01-26

**Authors:** Houda Zoubiri, Wassila Saiah, Amel Otmane, Hamza Saidi, Mohamed Makrelouf, Samir Ait Abderrhmane, Ali El Mahdi Haddam, Elhadj-Ahmed Koceir

**Affiliations:** 1 Bioenergetics and Intermediary Metabolism Team, Laboratory of Biology and Organism Physiology, Biological Sciences Faculty, Nutrition and Pathologies Post Graduate School, HouariBoumediene University of Sciences and Technology (USTHB), Bab Ezzouar, Algiers 16123, Algeria; houda.zoubiri@g.ens-kouba.dz (H.Z.); saiahbouchra@yahoo.fr (W.S.); saidi.hamza.dz@gmail.com (H.S.); 2 Biology and Physiology Laboratory, ENS Kouba, Algiers 16308, Algeria; 3 Biochemistry and Genetics Laboratory, University Hospital Center, Mohamed Lamine Debaghine, Bab El Oued, Algiers 16000, Algeria; dr.otmane.am@gmail.com (A.O.); mmakrelouf@gmail.com (M.M.); 4 Diabetology Unit, University Hospital Center, Mohamed Seghir Nekkache (ex. HCA de Aïn Naâdja), Algiers 16208, Algeria; saitabderrahmane@yahoo.fr; 5 Diabetology Unit, University Hospital Center, Mohamed Lamine Debaghine, Algiers I-University, Bab El Oued, Algiers 16000, Algeria; haddam25@yahoo.fr

**Keywords:** MAFLD (metabolic associated fatty liver disease), T2DM (type 2 diabetes mellitus), GLP-1 (glucagon-like peptide-1), adipokines, proinflammatory cytokines, metabolic syndrome, FLI (fatty liver index), IL-6 (Interleukine-6), IL-1β (Interleukine-1β), IL-17 (Interleukine-17), TNFα (tumor necrosis factor-alpha)

## Abstract

The triad association among type 2 diabetes mellitus (T2DM), metabolic associated fatty liver disease (MAFLD), and incretin secretion dysfunction, including GLP-1 (glucagon-like peptide-1) secretion dysfunction, maintains a critical cardiovascular risk and liver-related mortality. The aim of this study is to establish interactions between the GLP-1 plasma levels and metabolic syndrome clusters and adipokines profile (leptin, adiponectin, resistin) and proinflammatory cytokines (TNFα, IL-6, IL1β, IL-17) in diabetic subjects with or without MAFLD. The data revealed that insulin resistance (HOMA-IR) is present in all groups. MAFLD is more common in men than in women. The average FLI score in group IV was ≥70, confirming the diagnosis of MAFLD. The disorder of GLP-1 secretion is more pronounced in women than in men. HOMA-IR is negatively associated with plasma GLP-1 depletion in the MAFLD, T2DM, and MAFLD + T2DM groups. Adiponectin levels are decreased in all groups, as for GLP-1. In contrast, leptin, resistin, TNFα, IL-6, IL-1β, and IL-17 levels show an inverse correlation with GLP-1. GLP-1 accurately reflects metabolic and inflammatory status in subjects with MAFLD, T2DM, and diabetes—steatosis. The applied multivariate linear regression model confirms a highly significant association between MAFLD and GLP-1. It appears that plasma GLP-1 can be considered as biomarker in MAFLD and T2DM related to sex-gender disparities. Longitudinal studies are required to confirm these data.

## 1. Introduction

According to the World Health Organization, approximately 415 million adults aged 20 to 79 years are affected by T2DM, and this number is expected to increase to 800 million by 2040; whether in adult subjects [1] or adolescents and children [2]. In 2020, an international hepatic consensus suggested modifying the classification of hepatic steatosis to include both non-alcoholic fatty liver disease (NAFLD) and metabolic-associated fatty liver disease (MAFLD), and recently MASLD (metabolic dysfunction-associated steatotic liver disease) [3]. Thus, MAFLD has been proposed as an overarching concept to replace NAFLD [4]. In a large NHANES III study (National Health and Nutrition Examination Survey); patients diagnosed with MAFLD had a higher incidence of metabolic complications, including type 2 diabetes and hypertension [5]. Since this update, the superiority of MAFLD to NAFLD is still unclear, and further studies are needed. Nevertheless, it is important to report that previous studies that have highlighted that NAFLD or MAFLD is one of the most common chronic liver diseases, affecting approximately 25% of the global population to the extent that they require treatment [6]. MAFLD is a progressive disease leading not only to fatal hepatic complications, but also to extrahepatic complications such as cardiovascular diseases, chronic kidney disease, and cancers [7]. The term NAFLD was coined by Jürgen Ludwig et al. in 1980, following extensive research on this new liver disease [8]. In 2026, a meta-analysis confirmed a strong association between MAFLD, MASLD, and their long-term clinical evolution on vital prognosis in cardiovascular and cancerous pathologies [9]. However, MAFLD is now diagnosed as liver steatosis, visible on imaging or pathogenic by biopsy, without any secondary factors leading to hepatic fat accumulation, such as excessive alcohol consumption and prolonged use of steatogenic drugs [10]. Previous studies have shown that many factors are involved in the development and progression of NAFLD, including the hs-CRP/HDL-C ratio [11], insulin resistance [12], genetic factors [13], oxidative stress [14], and the gut microbiome [15]. These clinical studies highlight the differences in heterogeneity of phenotypes between MAFLD and NAFLD [16] and molecular mechanisms [17]. In 2021, approximately 1.27 billion MAFLD cases were reported globally [18], and the mortality risks from hepatic complications of MAFLD have been clearly distinguished from extrahepatic causes [19]. On the other hand, several meta-analyses have highlighted the role of incretins in T2DM pathophysiology [20], and recently with MAFLD [21]. Incretins’ history begins in 1902 with the discovery of secretin by Bayliss and Starling, as well as the endocrine role of the intestine [22]. An incretin is an intestinal hormone that stimulates insulin secretion in response to food intake, and this in proportion to the plasma glucose level; defined as a specific incretin physiological action [23,24]. Among the incretin family members, GLP-1 remains the most commonly used incretin in glycemic control of diabetic patients and has been largely studied for its multiple physiological enterohormonal effects [25]. Pharmacological agents targeting incretins are currently recognized as new anti-diabetic therapeutic classes, such as liraglutide, semaglutide, and exanatide, and are used to treat in diabetes [26], including gestational diabetes [27], as well as MAFLD [28,29]. GLP-1 is produced via the gene encoding proglucagon and is secreted by duodeno-jejunal enteroendocrine L cells. It is released in response to the ingestion of nutrients, particularly carbohydrates and monounsaturated lipids. The half-life of plasma GLP-1 is very short (2 min), as it is rapidly inactivated by dipeptidyl peptidase IV (DPP-IV) [30]. The GLP-1 binds to specific heptahelical cell receptors coupled with adenylate kinase activation, which stimulates cyclic AMP synthesis via the glucose-dependent protein kinase A signaling pathway. However, it should be emphasized that there is a duality in the mechanism of action between GLP-1 and GIP [31]. GLP-1 receptors agonists are expressed in different tissues, such as the pancreas, liver, heart, lung, adipose tissue, smooth muscle, kidney, and hypothalamus [32]. GLP-1 exerts several physiological effects, including satiety, by slowing gastric emptying and potentiates postprandial insulin secretion, with more than 70% of insulinotropic effects without hypoglycemia [33,34]. GLP-1 has no direct effect on insulin secretion in the absence of glucose and inhibits glucagon release [35]. The relationships among T2DM, MAFLD, and incretins are not well described in the literature or are often separately elucidated. Indeed, several studies have independently described associations between T2DM and MAFLD [36], or T2DM and GLP-1 [37], or MAFLD and GLP-1 [38]. These associations have most often been linked to hepatic insulin resistance [39] and cardiometabolic syndrome clusters [40]. All published meta-analyses support interactions between MAFLD and GLP-1 in T2DM. The hyperactivity of visceral adipose tissue has been recognized as a pivot point between MAFLD and adipocytokines [41]. This relationship is found between liver ectopic fat and GLP-1 in T2DM. These interactions are implicated in the genesis of metabolic disorders, such as metabolic syndrome, related to hepatic dysfunction and vasculo-inflammatory disorders [42]. MAFLD and T2DM are linked to the gut-liver axis, predominantly by inappropriate GLP-1 flux secretion [43], including the microbiota [44]. Several meta-analyses have been published to clarify the physiological mechanisms involved in the complex action of GLP-1 via the anatomical-physiological axis, such as entero-insular, entero-hepatic, entero-adipose tissue, entero-cerebral, entero-cardiovascular, and the immuno-inflammatory system [45,46,47,48,49,50]. On the other hand, the dysglycemia seen in patients with both T2DM and steatotic MAFLD is primarily linked to members the adipokine family, such as adiponectin [51] expression induced by incretin therapy, particularly GLP-1 [52]. Several studies have shown that leptin interacts with GLP-1 and its antagonist to reduce overeating. Eating behavior modulation by GLP-1 via neuronal leptin receptors is observed in the dorsomedial hypothalamus [53]. In the brain, LepR receptors were detected in GLP-1R-expressing neurons of the nucleus of the solitary tract, and leptin stimulated these neurons [54]. Previous studies using glucagon-like peptide-1 receptor agonists (GLP-1 RAs) as therapy have reported an elevation in adiponectin levels after GLP-1 RA injection [55,56]. In addition, dysregulated adipokine secretion in MAFLD and T2DM is strongly associated with the inflammation process, activated by the pro-inflammatory cytokine signaling pathway, which becomes exacerbated with significant production of TNFα (tumor necrosis factor alpha) and interleukins, particularly IL-6 [57,58]. Among the transitional interleukins, some studies have shown that interleukin-17 (IL-17) is involved in the pathogenesis of liver fibrosis and subsequent steatohepatitis [59,60]. IL-1β promotes hepatic inflammation by acting on liver sinusoidal endothelial cells via ICAM-1 (intercellular adhesion molecule 1) expression. In association with IL-6 and TNFα, IL-1β activates local immune cells and attracts other leukocytes to the liver, causing a chronic inflammatory state [61]. Finally, IL-1β contributes to the progression of hepatic inflammation to liver fibrosis via hepatic inflammasome activation [62]. On the other hand, previous work has demonstrated that TNFα infusion results in a reduction in GLP-1 synthesis, indicating that the inflammation state counteracts the beneficial effects of GLP-1 [63,64]. This study has a dual objective: (i) to determine the pleiotropic effects of GLP-1 as a modulator of adaptive physiological responses to metabolic syndrome cardiovascular risk factors and to the pro-inflammatory plasma adipocytokine profile; and (ii) to evaluate GLP-1 secretion and determine whether this can be considered as a biomarker for the prevention of MAFLD and T2DM in relation to sex-gender disparities.

## 2. Results

### 2.1. Patient Characteristics Specific to Different Pathological Conditions

Our study cohort included 590 participants divided into the following groups according to the inclusion and exclusion criteria: (i) 94 metabolic dysfunction-associated fatty liver disease (MAFLD) without diabetes (T2DM); (ii) 222 T2DM without MAFLD; (iii) 174 MAFLD with T2DM comorbidity; and 100 healthy subjects. All participant cohort were classified according to age and sex, with a sex ratio of men/women = 0.57. In our study, MAFLD was predominant in male compared to female participants (60.9% versus 39.1%, respectively, *p* < 0.001). Concomitantly, most men are overweight or obese (89%), with a median age of 48 years in men and 44 years in women (Table 1).

In contrast, the prevalence of type 2 diabetes mellitus (T2DM) was higher among women than men (53% versus 47%, respectively, *p* < 0.001). However, women with MAFLD and metabolic syndrome (MetS) were more likely to become diabetic compared to men. In this investigation, the MAFLD participants exhibited moderate steatosis in group II, unlike in group IV, according to the Fatty Liver Index.

### 2.2. Assessment of Anthropometric Data

**Figure 1:**
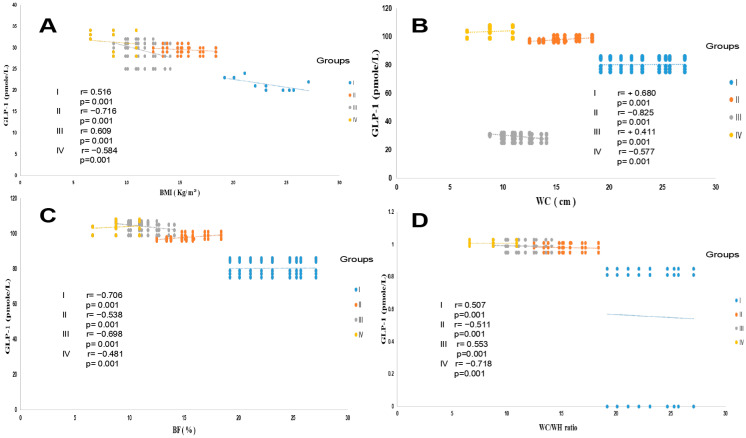
Pearson correlation between GLP-1 and BMI (**A**), WC (**B**), %BF (**C**), and the WC/WH ratio (**D**) in the different trial groups. Group I: healthy control participants; Group II: MAFLD participants; Group III: T2DM participants; Group IV: MAFLD + T2DM participants. MAFLD: metabolic associated fatty liver disease. T2DM: type 2 diabetes mellitus. GLP-1: glucagon-like peptide-1; BMI: body mass index; WC: waist circumference; WH: waist hips; %BF: body fat percentage. *p* < 0.001.

As summarized in Table 1, the anthropometric data showed a strong association between body mass index (BMI) and body fat percentage (%BF) in all groups. This correlation was not observed with body weight. The waist-to-hip ratio (WC/WH) ratio confirms that the diabetic (T2DM group) and diabetic steatosis (T2DM + MAFLD group) participants are obese, with android-type abdominal adiposity in men and gynoid-type abdominal adiposity in women, compared to the healthy group. Similarly, the %BF indicates that adipose tissue hypertrophy is exacerbated in the T2DM + NAFLD groups and to a lesser extent in the T2DM group. A positive and significant correlation was observed between GLP-1 and %BF in the MAFLD, T2DM, and MAFLD + T2DM groups (Figure 1C). The WC is correlated with insulinemia, which is increased by 61%, 78%, and 87% versus the healthy group (*p* < 0.01) in the MAFLD, T2DM, and MAFLD + T2DM groups, respectively (Table 2). Waist circumference (WC) revealed abdominal adipose tissue deposition, reflecting a significant increase in visceral adiposity (*p* < 0.001) in groups II (steatosis), III (diabetics), and IV (diabetic’s steatosis) compared to the healthy group. A positive and significant correlation was observed between GLP-1 and BMI (Figure 1A), between GLP-1 and WC (Figure 1B), and between GLP-1 and the WC/WH ratio (Figure 1D) in groups II, III, and IV compared to group I.

### 2.3. Data Comparing Metabolic Status and FLI Profile Across Groups

**Figure 2:**
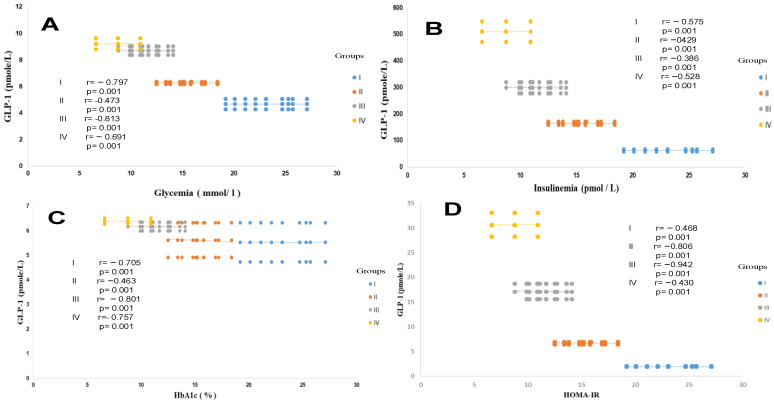
Pearson correlation between GLP-1 and glycemia (**A**), HbA1_C_ (**B**), insulinemia (**C**), and HOMA-IR index (**D**) in the different trial groups. Group I: healthy control participants; Group II: MAFLD participants; Group III: T2DM participants; Group IV: MAFLD + T2DM participants. MAFLD: metabolic-associated fatty liver disease. T2DM: type 2 diabetes mellitus. GLP-1: glucagon-like peptide-1. HOMA-IR: homeostasis model assessment-insulin resistance. *p* < 0.001.

**Figure 3:**
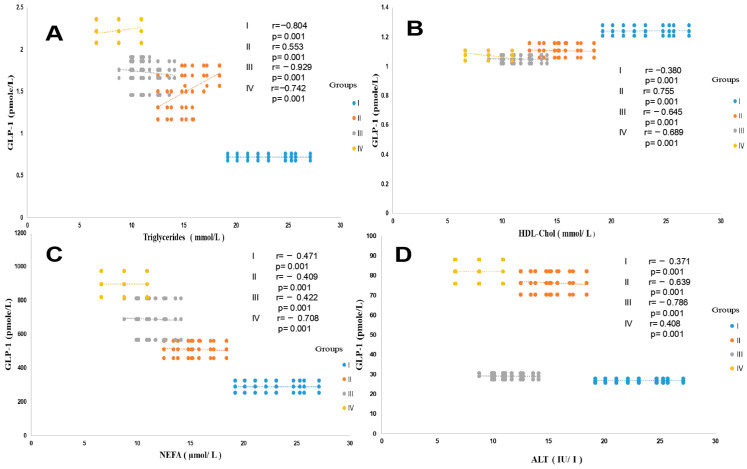
Pearson correlation between GLP-1 and triglycerides (**A**), HDL-cholesterol (**B**), NEFA (**C**), and ALT (**D**) in the different trial groups. Group I: healthy control participants; Group II: MAFLD participants; Group III: T2DM participants; Group IV: MAFLD + T2DM participants. MAFLD: metabolic-associated fatty liver disease. T2DM: type 2 diabetes mellitus. GLP-1: glucagon-like peptide-1. NEFA: non-esterified fatty acids. ALT: alanine aminotransferase. *p* < 0.001.

Similarly, the HOMA-IR index evolves in parallel with hyperinsulinism. The HOMA test is used as a screening tool to detect MAFLD in a diabetic subject [65]. Acute insulin resistance is revealed in group IV (+93% vs. control groups, *p* < 0.001) and in the T2DM and MAFLD groups (+88% and +70%, respectively, vs. control subject). Referring to the National Cholesterol Education Program Adult Treatment Panel III (NCEP/ATP-III) consensus [66], the MAFLD group manifests glucose intolerance, but not hyperglycemia. Conversely, the T2DM and MAFLD + T2DM groups are characterized by hyperglycemia (plasma glucose level > 7 mmol/L, with HbA1c < 7%; Table 2), which indicates adequate glycemic control. We found an inverse correlation between GLP-1 and glycemia, insulinemia, HbA1C, and HOMA-IR in the MAFLD and T2DM groups (Figure 2A–D). In this study, we observed a strong association between severe dyslipidemic triglyceridemia and cholesterolemia, with elevated LDL-c and decreased HDL-c, in groups I, II, III, and IV versus the control group (Table 2). Dyslipidemia was especially due to NEFA hepatic infiltration, with NEFA reaching paroxystic levels in group IV versus group I (Table 2). In addition, the hepatic lipids observed in histopathological MAFLD diagnosis increase together with serum triglyceride accumulation. This is obviously indicative of severe liver deterioration in the diabetic steatosis groups versus the healthy group. We recorded a positive correlation between hypertriglyceridemia and serum NEFA levels in the MAFLD + T2DM group (Figure 3A,B). On the other hand, the correlation is negative between serum HDL-cholesterol levels and GLP-1 concentrations in this same group (Figure 3B). No correlation was observed between GLP-1 and serum total cholesterol levels.

#### Fatty Liver Index (FLI) Profile

**Figure 4:**
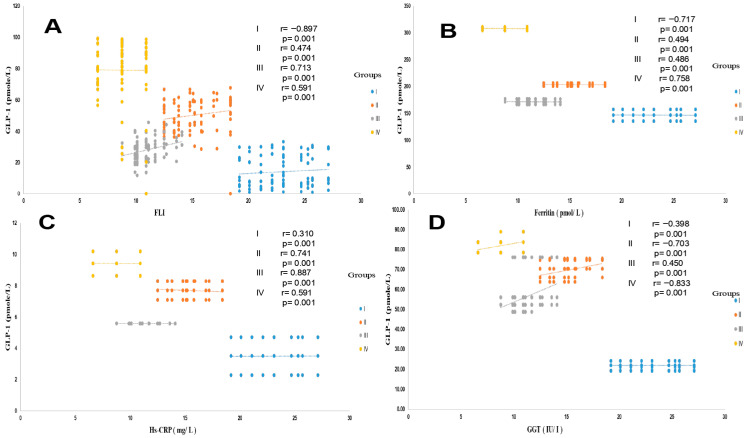
Pearson correlation between GLP-1 and FLI (**A**), ferritin (**B**), Hs-CRP (**C**), and GGT (**D**) in the different trial groups. Group I: healthy control participants; Group II: MAFLD participants; Group III: T2DM participants; Group IV: MAFLD + T2DM participants. MAFLD: metabolic-associated fatty liver disease. T2DM: type 2 diabetes mellitus. GLP-1: glucagon-like peptide-1. GGT: gamma glutamyl transferase. FLI: fatty liver index. *p* < 0.001.

As shown in Table 2, the mean FLI value was significantly higher in group IV (MAFLD + T2DM) compared to the MAFLD, T2DM, and healthy groups (81%, 66%, and 40%, respectively). However, the massive increase in MAFLD was only observed in group IV. According to the FLI value, MAFLD is moderate in group II, although GGT and ALT levels are elevated compared to group I. A highly significant positive correlation was found between the FLI value and serum triglyceride and GGT levels (r = +0.874 and r = +0.761, respectively, *p* < 0.001). FLI was also positively correlated with BMI and waist circumference (r = +0.658 and r = +0.593, respectively, *p* < 0.001). We observed a highly significant association between FLI and HOMA-IR index. Conversely, the FLI profile is inversely correlated with GLP-1 secretion levels, particularly in group IV (Figure 4A). Similarly, FLI is positively correlated with pro-inflammatory cytokine levels, particularly TNFα and IL-6, in group IV (r = +0.409 and r = +0.371, respectively, *p* < 0.001). Conversely, no association was observed between FLI and serum leptin or total cholesterol levels.

Taking into account the complexity of the metabolic syndrome requires controlling for confounding variables, such as BMI, age, sex, and T2DM duration as covariates; a multivariate linear regression analysis was established to investigate whether MAFLD can prevent changes in GLP-1 secretion levels (Table 3). After adjustment for age and sex, GLP-1 was inversely associated with the presence of MAFLD and/or T2DM (standardized β = −0.633, *p* < 0.001). This association remained significant after further adjustment for BMI (standardized β = −0.647, *p* < 0.001). After additional adjustment for T2DM duration, the association was attenuated but remained statistically significant (standardized β = −0.283, *p* < 0.001). Similarly, we observed a significant association after further adjustment for sex-gender (standardized β = −0.688, *p* < 0.001).

The data presented in Table 2 show that liver function is severely impaired in the MAFLD + T2DM group. The serum ALT, AST, and GGT concentrations are abnormally high compared to the control group, up to three to four times the normal value. The hypertriglyceridemia observed in the MAFLD and MAFLD + T2DM groups is positively correlated with serum GGT levels, as a sign of liver necrosis without non-alcoholic steatohepatitis (Figure 4D). This is also confirmed by the positive correlation between decreased plasma GLP-1 levels and enhanced FLI, with concomitant high elevation of serum ferritin levels in the MAFLD and MAFLD + T2DM groups and normal serum ferritin levels in the T2DM group (Figure 4A,B). Hepatic steatosis often manifests as an elevation of transaminases ALT and AST; however, ALT is generally more specific to non-alcoholic liver steatosis. Interestingly, the increase in ALT levels is positively associated with the drastic fall in serum GLP-1 levels in steatosis patients in groups II and IV (Figure 3D). Furthermore, in the MAFLD + T2DM group, it is noted that liver dysfunction is associated with an acute inflammatory state and excessively high Hs-CRP concentrations. Concomitantly, plasma ferritin and total bilirubin concentrations are exacerbated in groups II and IV (Table 2). A strong correlation was found between ALT levels and Mets clusters in the MAFLD + T2DM group. An association was observed between decreased GLP-1 and visceral adiposity (WC), HOMA-IR, and hypertriglyceridemia. A blood pressure disorder was observed in group IV compared to participants in the other groups, who remained normotensive. However, if we consider the NCEP-ATP III criteria (PAS > 130 mm Hg), a blood pressure disorder seems to occur in the MAFLD + T2DM group.

### 2.4. Adipokines/Pro-Inflammatory Cytokines and GLP-1 Profile Analysis

#### 2.4.1. Plasma Glucagon-like Peptide 1 (GLP-1) Profile

Plasma GLP-1 production is presented in Table 2. It should be remembered that GLP-1 was measured after a 12-h fasting phase, followed by consumption of a meal containing 820 kcalories. The data shown in Table 2 represent the average values between 60 and 15 min (Δ GLP-1) after the meal digestion phase. Fasting and fed state values vary between 11.7 ± 1.71 and 34.5 ± 3.81 pmole/L, respectively, in the control group. It should be noted that GLP-1 secretion rates were decreased in both the fasting and fed states in the steatotis and diabetic groups compared to the healthy group (Table 2). We recorded decreases of 19%, 25%, and 76% and of 40%, 58%, and 68%, respectively in groups II, III, and IV (*p* < 0.001). Remarkably, GLP-1 secretion was significantly increased in the postprandial (PP) state compared to the fasting (Fs) state in all groups. GLP-1 synthesis was decreased by 66%, 54%, 40%, and 39% in groups I, II, III, and IV, respectively (*p* < 0.001). However, when considering each nutritional state separately, GLP-1 production decreased in both MAFLD and T2DM participants compared to the control group (40% to 19% decrease, respectively, *p* < 0.001). GLP-1 depletion was accentuated when steatosis was associated with diabetes in the MAFLD + T2DM group. GLP-1 concentrations are reduced by 43% and 68%, respectively, in the fasting and postprandial states. In the MAFLD + T2DM group, we found a negative correlation between plasma GLP-1 levels and HbA1c (Figure 2C), HOMA-IR (Figure 2D), hypertriglyceridemia (Figure 3A), WC (Figure 1B), and Hs-CRP (Figure 4C). Similarly, fasting GLP-1 values were inversely correlated with fasting insulin values in groups II, III, and IV compared to the control group (Figure 2B). It is important to note that, despite the drop in plasma GLP-1 levels in the steatosis participants (Group II), glucose intolerance was not affected in this group. Group II remains normoglycemic, although it presents hyperinsulinism (Table 2).

#### 2.4.2. Plasma Adipokine Profile

##### Plasma Leptin Levels

**Figure 5:**
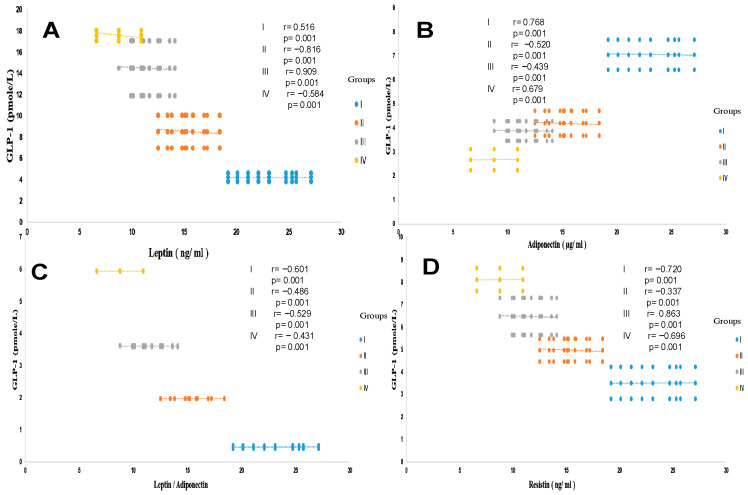
Pearson correlation between GLP-1 and leptin (**A**), adiponectin (**B**), leptin-adiponectin ratio (**C**), and resistin (**D**) in different trial groups. Group I: healthy control participants; Group II: MAFLD participants; Group III: T2DM participants; Group IV: MAFLD + T2DM participants. MAFLD: metabolic-associated fatty liver disease. T2DM: type 2 diabetes mellitus. GLP-1: glucagon-like peptide-1. *p* < 0.05; *p* < 0.01; *p* < 0.001.

The leptin profile is sex-dependent. In this study, the data reveal that women have higher average serum leptin levels than men in the control group (5.22 ± 0.40 versus 3.25 ± 0.43 ng/mL, respectively). The sex difference is highly significant, at 60% (*p* < 0.001). This sex variation is maintained in the steatosis, diabetic, and diabetic-steatosis groups. Leptin secretion increases depending on the intensity of adipose tissue accumulation (% fat and BMI). Surprisingly, the data obtained on adipokine status shows that leptin levels are higher proportionally to the fat mass increase, especially in the diabetic-steatosis group, and are even elevated in the diabetic group compared to the control group. We recorded an average increase of 45%, 55%, and 71% in groups II, III, and IV, respectively (Table 4). A strong correlation was observed between plasma GLP-1 levels and leptinemia in the MAFLD, T2DM, and MAFLD + T2DM groups (Figure 5A). Similarly, the correlation is positive between leptinemia and HOMA-IR in the three groups (r = +0.88; r = +0.91; r = +0.99, respectively, *p* < 0.001).

##### Plasma Adiponectin Levels

Similar to the leptin profile, adiponectin secretion was also sex-dependent. A significant difference was observed between average circulating serum adiponectin levels in women and men (33% increase in women), which were 8.07 ± 1.15 versus 6.04 ± 0.12 ng/mL in the control group (Table 4). In contrast to leptin, plasma adiponectin concentrations were reduced in all MAFLD, T2DM, and MAFLD + T2DM participants, resulting in an elevated leptin/adiponectin (L/A) ratio (Figure 5C). In addition, we noted an association between adiponectin depletion levels and a reduction in circulating GLP-1 concentrations in the MAFLD + T2DM group (Figure 5B). In addition, a positive correlation is observed between adiponectin and GLP-1 levels in the MAFLD and T2DM groups (Figure 5B). In contrast, adiponectin levels are inversely correlated with BMI, insulin levels, serum triglycerides, and insulin resistance in the T2DM group (r = −0.45, r = −0.39, r = −0.51, r = −0.69, respectively, *p* < 0.001). Furthermore, a negative correlation is observed in the MAFLD + T2DM (r = −0.44, r = −0.38, r = −0.50, r = −0.68, respectively, *p* < 0.001) group. However, we did not find a correlation between adiponectin levels and % BF in groups II, III, and IV. Unlike hyperleptinemia, it appears that hypoadiponectinemia is an independent factor in the evolution of body fat mass during the steatosis phase, except in diabetic participants, where the correlation is positive.

##### Plasma Leptin/Adiponectin Ratio

We observed a correlation between leptin/adiponectin (L/A) ratio and plasma GLP-1 level (Figure 5C). Furthermore, the L/A ratio was negatively correlated with HDL-cholesterol levels (r = −0.314, *p* < 0.001). Conversely, the L/A ratio was very weakly correlated with LDL-cholesterol levels (r = 0.117, *p* < 0.001), with no significant correlation between the L/A ratio and serum NEFA level (r = 0.066, *p* = 0.053). Overall, the L/A ratio was also correlated with the ratio of total cholesterol to HDL-cholesterol (Table 4). Regarding the other parameters, the L/A ratio was weakly correlated with glycated hemoglobin A1c, FLI, and GGT, and there was no correlation between L/A ratio and AST. On the other hand, the L/A ratio was changes proportionally with increased leptin concentrations and reduced adiponectin levels in the diabetic groups, but not in the steatosis groups, compared to the control group (Figure 5B). This leads to an elevated L/A ratio with acute steatosis when associated with diabetes (group IV). The L/A ratio showed positive correlations with BMI (r = 0.588, *p* < 0.001) and % BF (r = 0.603, *p* < 0.001).

##### Plasma Resistin Levels

Unlike leptin and adiponectin plasma levels, resistin production is not sex-dependent. The average resistin plasma levels are slightly elevated in men compared with women, but the difference was non-significant (Table 4). For both women and men, a mean value of 3.52 ± 0.24 ng/mL was obtained in group I; 5.16 ± 0.29 ng/mL in group II; 6.48 ± 0.38 ng/mL in group III; and 7.67 ± 0.29 ng/mL in group IV. Plasma resistin levels were increased in all diabetic subjects with or without steatosis. It is noted that resistinemia tends to increase during the phases of steatosis, diabetes, and the steatosis-diabetes association. Serum resistin levels are highly elevated in the MAFLD-T2D group. We recorded increases of 29%, 42%, and 64%, respectively, in the MAFLD, T2DM, and MAFLD + T2DM groups. The difference was highly significant (*p* < 0.001). Resistinemia evolved concomitantly with insulin resistance. In this study we observed a strong positive correlation between plasma resistin levels and HOMA-IR in groups II and IV (r = 0.803, r = 0.776, respectively, *p* < 0.001). Interestingly, GLP-1 concentrations were inversely correlated with resistin concentrations in the MAFLD, T2DM, and MAFLD + T2DM groups (Figure 5D).

#### 2.4.3. Plasma Pro-Inflammatory Cytokine Profile

##### Plasma TNFα (Tumor Necrosis Factor-Alpha) Levels

**Figure 6:**
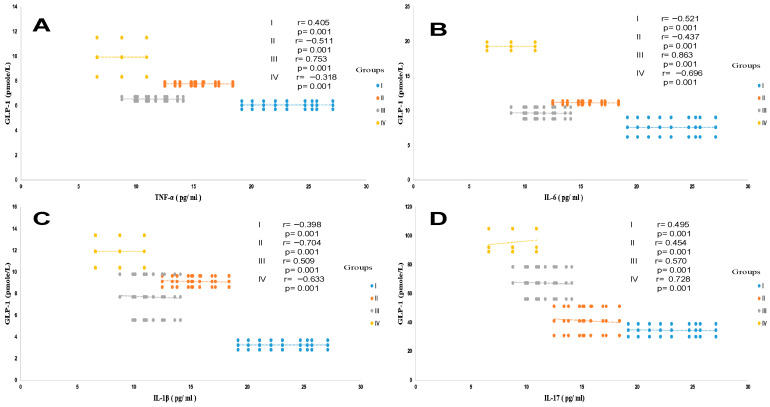
Pearson correlation between GLP-1 and TNFα (**A**), IL-6 (**B**), IL-1β (**C**), and IL-17 (**D**) in the different trial groups. Group I: healthy control participants; Group II: MAFLD participants; Group III: T2DM participants; Group IV: MAFLD+ T2DM participants. MAFLD: metabolic-associated fatty liver disease. T2DM: type 2 diabetes mellitus. GLP-1: glucagon-like peptide-1; TNFα: tumor necrosis factor-alpha; IL-6: interleukin-6; IL-1β: interleukin-1 β, IL-17: interleukin-17. *p* < 0.05; *p* < 0.01; *p* < 0.001.

In this study, we observed that plasma TNFα levels are increased proportionally to the elevation of serum Hs-CRP levels in all diabetic and steatosis groups compared to healthy subjects. However, the TNFα plasma levels are highly deleterious in the steatosis group (groups II and IV) and to a lesser extent in the non-steatosis diabetic group (group III). We observed a positive correlation between Hs-CRP levels and TNFα, IL-6, IL-1β, and IL-17 in the MAFLD + T2DM group (r = +0.68; r = +0.77; r = +0.91; r = +0.51, respectively, *p* < 0.001). We found that TNFα is increased by 39% and 22% (*p* < 0.001) in the MAFLD + T2DM and MAFLD groups, respectively (Table 5); whereas, TNFα is increased only by 6% in the diabetic group without steatosis versus the healthy group. The correlation is positive between TNFα, IL-6, IL-1β, and BF% in the MAFLD (r = +66, r = +47, r = +38, respectively, *p* < 0.001) and MAFLD + T2DM (r = +67, r = +49, r = +55, respectively, *p* < 0.001) groups. In contrast, plasma GLP-1 is inversely correlated with TNFα, IL-6, IL-1β, and IL-17 concentrations in the T2DM and MAFLD + T2DM groups (Figure 6A–D).

##### Plasma IL-6 (Interleukin-6) Levels

Unlike the TNF alpha plasma profile, which was increased only in the steatosis groups (groups II and IV), plasma IL-6 levels were elevated in the steatosis + diabetic group (group III). The inflammation due to interleukin-6 (IL-6) is more fulminating than that due to TNFα (Table 5; Figure 6B). Interestingly, MAFLD groups III and IV exhibited IL-6 increases of 60% and 34% versus the healthy group (19.3 ± 0.56 and 11.6 ± 0.23 vs. 7.62 ± 1.43 pg/mL; *p* ≤ 0.001, respectively). Furthermore, we found a positive correlation between IL-6 levels and L/A ratio in groups II and IV (r = 0.133, *p* < 0.001 and r = 0.130, *p* < 0.001, respectively), whereas there was no correlation between IL-6 level and adiponectin level in the steatosis groups (II, IV). It is important to highlight the differences in the pro-inflammatory effects between TNFα and IL-6 in MAFLD and its progression to liver damage (NASH, cirrhosis). In our study, we observed that IL-6 levels show a stronger correlation with MAFLD severity, as indicated by high FLI values (r = +0.86, *p* < 0.001), than with TNFα level, which showed only a moderate correlation (r = +0.33, *p* < 0.01).

##### Plasma IL-1β (Interleukin-1beta) Levels

We showed a strong increase in IL-1β by 72% and 64% in the MAFLD + T2DM and MAFLD groups, respectively; *p* < 0.001 versus the healthy group. IL-1β promotes hepatic steatosis by stimulating the accumulation of triglycerides in steatosis groups II and IV (Table 5). We established a positive correlation between IL-1β levels and triglyceride levels in groups II and IV (r = 0.545, *p* < 0.001 and r = 0.308, *p* < 0.001, respectively). Likewise, there was strong correlation between IL-1β level and plasma NEFA (non-esterified fatty acids) level in steatosis groups II and IV (r = 0.661, *p* < 0.001 and r = 0.801, *p* < 0.001, respectively). It appears that IL-1β, together with IL-6 and TNFα, activates lipolysis by releasing a significant flux of NEFA. The correlation is positive and highly significant in steatosis groups II and IV (*p* < 0.001). On the other hand, IL-1β contributes to steatosis progression from fatty liver to inflammatory liver fibrosis, as evidenced by elevated plasma ferritin and hs-CRP levels in steatosis groups II and IV. The difference is highly significant (*p* < 0.001) compared to the control group.

##### Plasma IL-17 (Interleukin-17) Levels

We have seen a clear increase in IL-17 by 62% and 16% in the MAFLD + T2DM and MAFLD groups, respectively; *p* < 0.001 versus healthy group. Our study confirms the importance of IL-17 in steatosis group III and diabetic-steatotic participants (group IV), in whom MAFLD is complicated with presence of diabetes (Table 5). Indeed, we observed a positive correlation between enhanced IL-17 levels and increased plasma NEFA levels (r = 0.851 and r = 0.791, *p* < 0.001, respectively). Furthermore, we observed a proportional and synergistic increase in transaminase activities, particularly the AST/ALT ratio, with the dramatic rise in IL-17 levels in group IV. Surprisingly, we noticed that, in group IV, concomitantly with the increase in IL-17 and the increase in plasma NEFA levels, insulin sensitivity decreased significantly, as demonstrated by the increase in HOMA-IR index, which leads to insulin resistance.

### 2.5. An Integrated Analysis Relating All Factors Within Each Pathological Condition

#### 2.5.1. Data Analysis for T2DM Patients

The cardiovascular risk factors of metabolic syndrome are found in all patients in group III, in both men and women. GLP-1 secretion appears to be decreased, particularly in women compared to men. However, It is important to emphasize that the secretion rates of GLP-1 are equivalent in group I (Table 2), whether in women or men. In this study, hypertriglyceridemia is more pronounced in men than in women. This is concomitant with elevated GGT and resistin in men, and slightly less so in women. Inflammation is found in both sexes, characterized by elevated pro-inflammatory cytokines (TNFα, IL1β, IL-6, and IL-17). Abdominal adiposity (waist circumference) is more pronounced in men compared to women. This is concomitant with increased leptin levels and decreased adiponectin, which appears to affect the control of food intake (see discussion section related to leptin’s effect on the hypothalamic nuclei).

#### 2.5.2. Data Analysis for MAFLD Patients

GLP-1 secretion dysfunction is significantly reduced in female patients compared to men, but glucose tolerance is preserved, with patients remaining normoglycemic. According to the NCEP-ATPIII criteria (3/5 criteria), metabolic syndrome is not observed. Based on FLI data, female patients in this group appear less susceptible to developing massive steatosis compared to men. Significantly elevated GGT levels and FLI were positively correlated with waist circumference and hypertriglyceridemia in male patients compared to female patients. Adipocytokine disturbances indicate that inflammation is more acute in men compared to women (see discussion section).

#### 2.5.3. Data Analysis for T2DM-MAFLD Patients

All the metabolic syndrome clusters are found in group IV, whether in women or men. All diabetic patients in group IV and group III have uncontrolled diabetes, if we refer to the HbA1c limit levels, which should be <7% (9.21 ± 0.42%; 8.71 ± 0.33%, *p* < 0.001, respectively). GLP-1 secretion appears to continue to decline in female patients compared to male patients. Conversely, according to the FLI, the degree of MAFLD is acute in men versus women. This is positively correlated with the degree of inflammation, reflected by pro-inflammatory cytokines, compared to women. The drop in adiponectin levels is proportionally correlated with GLP-1 secretion decrease in all patients in this group, concomitant with increased insulin resistance (HOMA index).

## 3. Discussion

Our clinical data divulge important abnormalities of GLP-1 secretion in T2DM participants with or without MAFLD. Plasma GLP-1 levels progressively decreased in participants with steatosis and diabetes without comorbidity, and in diabetic steatotic participants with comorbidity. GLP-1 secretion in the different groups of participants can be classified into three stages: moderate in MAFLD without diabetes (group II), severe in T2DM without steatosis (group III), and morbid in diabetic steatosis (group IV). In our study, five factors modulated GLP-1 secretion or bioavailability: (i) insulin resistance; (ii) liver dysfunction; (iii) visceral adipose tissue hyperactivity; (iv) adipokine profile; and (v) pro-inflammatory cytokine production.

(i) The first major point is linked to the MAFLD—T2DM—GLP-1 plasma level interaction and metabolic syndrome clusters, including FLI.

It is undeniably confirmed that the incretin effect of GLP-1 is inhibited in the presence of insulin resistance (HOMA-IR). The insulin resistance observed in steatosis without diabetes (MAFLD group) is explained by hepatic insulin resistance due to highly active lipogenesis, marked by increased de novo fatty acid synthesis (malonyl CoA pathway), which stimulates overproduction of VLDL lipoproteins [67]. Hepatic insulin resistance is also due to an exacerbated increase in adipocyte lipolysis, leading to intrahepatocyte lipid infiltration [68]. Gradually, this fatty infiltration affects the skeletal muscle when steatosis becomes complicated by diabetes (MAFLD + T2DM group). At this stage, insulin resistance becomes hepato-muscular, and there is an alteration of the insulin signaling pathway via activation of the protein kinase C epsilon pathway [69].

Our data are explained by the involvement of adipose tissue via triglyceride dyslipidemia, which we found in the steatosis groups with and without diabetes. Indeed, lipolysis of visceral adipose tissue (VAT), which is highly hypertrophied (WC, WC/WH ratio, BF percentage) and rich in adrenergic receptors, particularly when steatosis is associated with obesity [70], releases an excessive flow of triglycerides, creating a large pool of NEFAs, which maintains insulin resistance [71]. In addition, adipocyte triglyceride lipase hyperactivity and inhibition of NEFA oxidation partly explain the glucose intolerance of steatosis subjects (MAFLD group) and those with diabetic hyperglycemia (T2DM group) [72].

In the MAFLD and T2DM groups, hypertriglyceridemia is often found, associated with an increase in plasma levels of NEFA, which infiltrates into the liver, leading to hepatic steatosis, particularly in group IV. This explains the strong increase in FLI. During the development of hepatic steatosis, the lipids that accumulate in the liver come not only from the plasma NEFA pool (resulting from adipose tissue lipolysis), but also from newly synthesized fatty acids from glucose via de novo hepatic lipogenesis (malonyl CoA pathway). In our study, groups III and IV exhibit hyperinsulinism associated with insulin resistance. The literature states that insulin activates the expression of liver genes necessary for lipid synthesis via the transcription factor SREBP-1c (sterol regulatory element-binding protein). SREBP-1c plays a crucial role in liver steatosis development. Indeed, mice with SREBP-1c knockout do not develop hepatic steatosis, unlike control mice (in which expression of SREBP-1c lipogenic genes is conserved). This study was corroborated by another study which showed that the transcription of lipogenesis genes, including SREBP-1c, was activated by the glucose-signaling pathway, and that this was primarily related to the transcription factor ChREBP (carbohydrate responsive element-binding protein) [73].

The impaired blood glucose regulation observed in the MAFLD and T2DM groups is no longer mediated by incretins, given the lack of effect of GLP-1, a potent inhibitor of glucagon secretion that has been proposed as a therapeutic target [74]. Some studies have suggested that GLP-1 acts through paracrine effects by increasing somatostatin and insulin concentrations, thereby modulating steatosis in diabetic subjects [75]. Recently, it has been shown that elevated free fatty acid (FA) release induced by a high-fat diet, particularly saturated FA, but not unsaturated FA, leads to duodenal lipotoxicity that inhibits GLP-1 synthesis via the PPARdelta/UCP2 (peroxisome proliferator-activated receptor delta/UCP2 uncoupling protein 2) pathway [76]. These data explain the GLP-1 hypoincretinemia seen in MAFLD [77] and GLP-1 depletion in T2DM [78]. These studies found a strong association between GLP-1 depletion and dysfunction of transmembrane G proteins, particularly when they are activated by increased free fatty acids. This primarily involves GPR40 (G protein-coupled receptor 40) in beta cells. This dysregulation also affects other G proteins, such as GPR119 (G protein-coupled receptor 119) and GPR120 (G protein-coupled receptor 120). GLP-1R expression has also been described as poorly detectable (low mRNA concentration) in hepatocytes from liver biopsies of subjects with MAFLD and is undetectable in patients with NASH [79].

It is crucial to understand the cellular and molecular mechanisms by which GLP-1 (active or total) crosses the blood-brain barrier and exerts its physiological effects on hypothalamic neurons. Relevant meta-analyses and clinical studies have specified that GLP-1crosses the blood-brain barrier (BBB) primarily through specific areas where it is less strict, such as the circumventricular organs (area postrema, subfornical organ, median eminence, and choroid plexus), notably via hypothalamic tanycytes, which facilitate its entry. The tanycytes release VEGF (vascular endothelial growth factor), enhancing central access of GLP-1 (or its agonists). GLP-1 can also activate the vagus nerve to transmit signals to the brain. Transendothelial GLP-1 transport is active, involving the GLP-1 receptor (GLP-1R), similar to the leptin receptor, suggesting a mechanism dependent on a specific transporter for passage through BBB cells [80]. Other meta-analyses have shown that, in humans, GLP-1R is expressed throughout the cerebral cortex, with particularly high density in the ventromedial hypothalamus, paraventricular nucleus, and arcuate nucleus. The GLP-1R has also been detected in the hippocampus, thalamus, amygdala, caudate nucleus-putamen, and globus pallidus [81]. Based on all these studies, it appears that wide GLP-1R brain distribution suggests that GLP-1 may be involved in the regulation of multiple neurological and cognitive functions in addition to regulating glucose metabolism. It has been suggested that many GLP-1 effects are mediated indirectly via binding to GLP-1R in enteric or vagal sensory neurons. Indeed, GLP-1 activates afferent vagus nerve fibers, which transmit satiety signals to the hypothalamus, inducing downstream neuronal responses that activate brain circuits involved in regulating eating behavior. The study also demonstrated that GLP-1 may not use CSF (cerebrospinal fluid) to access the brain, but can cross the blood-brain barrier directly [82]. GLP-1 affects the brain by three mechanisms: (i) some GLP-1 is produced by neuronal cells located in the nucleus of the solitary tract of the brainstem; (ii) GLP-1 secreted by the gut can reach the central nervous system via the blood-brain barrier (area postrema, subfornical organs); and (iii) the afferent vagal nervous system is a mediator of the effects of GLP-1 on the brain; which explains the therapeutic targets of GLP-1 [83]. GLP-1 control of food intake is exerted via the activation of anorexigenic POMC (pro-opiomelanocortin)/CART (cocaine- and amphetamine-regulated transcript) neurons and inhibits orexigenic NPY (neuropeptide Y)/AgRP (agouti-related peptide) neurons in the arcuate nucleus. In hypothalamic neurons, the effects of GLP-1 are mediated via glucose metabolism-dependent inhibition of the AMPK (AMP-activated protein kinase) signaling pathway. GLP-1 can also interact with other anorexigenic signals, including leptin. Taken together, these data suggest that GLP-1 contributes to the negative energy balance by decreasing caloric intake and increasing energy expenditure [84].

Regarding the fatty liver index (FLI), our study demonstrated that the FLI value represents an essential diagnostic data point for MAFLD in patients with type 2 diabetes whose FLI score is ≥70, confirming the diagnosis of MAFLD (group IV). The data from this clinical investigation validate the ability of the FLI to identify MAFLD patients at risk of cardiovascular events [85]. In our study, we demonstrated the association between exacerbated FLI values and elevated plasma levels of NEFAs in group IV. This relationship explains the development of glucose intolerance and hepatic insulin resistance, which leads to the progression of steatotic patients towards a diabetic state [86]. The association between FLI and impaired insulin sensitivity is also confirmed by the strong correlations between the indirect markers of peripheral systemic insulin resistance, such as the triglyceride-glucose index [87], and positive relationships between FLI and the HOMA-IR index, markers of hepatic insulin resistance in MAFLD [88]. We have shown that FLI was significantly negatively correlated with adiponectin levels and positively correlated with leptin in the diabetic groups (III and IV). Several studies have shown that FLI is a risk factor for developing diabetes in a steatopic individual, independent of insulin sensitivity [89]. FLI predicts hepatic steatosis associated with MAFLD due to nutritional factors specific to lipids that link them, as GLP-1 secretion is lipid-dependent. Similarly, MAFLD is also lipid-dependent, making incretin-based therapies promising for treatment by improving MAFLD [90].

(ii) The second crucial point is the lied between MAFLD—T2DM—GLP-1 plasma level interactions and the plasma adipokine profile.

In both groups of diabetics with or without steatosis, we demonstrated hypoadiponectinemia concomitant with a marked depletion of plasma GLP-1 concentrations, with an increase in leptin and resistin. Hyperleptinemia does not appear to be due solely to overproduction of leptin by adipose tissue, as insulin resistance increases circulating leptin concentrations [91], but more specifically to a situation of resistance to the action of leptin, favored by drop in GLP-1 concentrations. Indeed, GLP-1 has been reported to maintain a state of satiety after food intake by regulating the secretion of leptin and ghrelin via the vagus nerve in diabetic patients [92].

This supports major interactions at the brain level between GLP-1 and leptin, as GLP-1 receptor (Glp-1R) and leptin are distributed in the hypothalamic regions (arcuate nuclei and solitary tract). Through this, GLP-1 and leptin exert their satiety-inducing effect. In our study, it appears that a lack of GLP-1 leads to an accumulation of leptin in the cerebrospinal fluid, which inhibits its passage from the blood to the brain via the blood-brain barrier (BBB). Indeed, leptin, transported in the cerebrospinal fluid (CSF), reaches the brain, more precisely in the hypothalamus at the arcuate nucleus, where it is recognized by specific receptors (Lep-Rb) and then internalized into neurons [93]. In the hypothalamic region, the selectivity function of the blood-brain barrier is ensured by glial cells, called tanycytes, irrigated by insulin-sensitive fenestrated blood vessels [94].

It is precisely in the tanycytes that leptin binds to and then accesses the neurons of the arcuate nucleus. In our study, we hypothesized a state of resistance to leptin penetration into the brain (arcuate nucleus), where leptin remains trapped in the tanycytes, which leads to its accumulation in the CSF, particularly in diabetes [95]. Consequently, it would also accumulate in the blood, which would explain our results. The originality of our study lies in the fact that GLP-1 deficiency could also be associated with this form of leptin resistance via the insulin resistance signaling pathway [96]. Based on an animal model, this set of events represents a brake on leptin signaling pathways at the hypothalamic level via inhibition of the JAK-STAT3 (Janus activated kinase-signal transducer and activator of transcription 3) signaling pathway [97].

The data obtained with adiponectin are concomitant with GLP-1 depletion, but also with insulin resistance. Several meta-analyses confirm that hypoadiponectinemia is most often associated with MAFLD and contributes to the onset of T2DM in a steatotic subject. However, the administration of a GLP-1 mimetic treatment corrects this disorder by increasing serum adiponectin levels [98]. One of the arguments explaining hypoadiponectinemia is linked to alteration of the same insulin, adiponectin, and GLP-1 signaling pathways related to diet-induced stetaosis [99]. These pathways are mediated by modulating the NF-kappaB p65/PI3K/Akt (nuclear factor erythroid 2-related factor 2 p65/phosphatidylinositol 3-kinase/AKR mouse T cell lymphoma or protein kinase B) signaling pathway [100].

Regarding resistin, to our knowledge, very few studies have examined the effects of resistin on GLP-1 secretion. Recently, a cross-sectional study examined the relationships among resistin, steatosis, and obesity. The results showed that resistin levels were elevated, while adiponectin levels were decreased. Treatment with thiazolidinediones (PPAR-γ receptor mimics) was associated with a decrease in resistin [101].

The results of our study showed that high levels of resistin were associated with high HOMAIR values and inversely proportional to GLP-1 levels. Regarding the literature, meta-analyses on resistin are controversial. Some authors do not consider it an adipokine, but rather a pro-inflammatory cytokine, given its synergy with interleukin-6 and its role correlated with visceral adipose tissue [102]. Resistin signaling pathways are not clearly understood. It appears that resistin uses the NF-kB (nuclear factor-kB) pathway in the liver, which explains its paroxysmal level in our study [103]. In the MAFLD subjects, an important study has highlighted an association between the genetic variant rs3745367 of RETN (the resistin gene) and the development of NAFLD confirmed by biopsy [104]. Interestingly, treatment with a GLP-1 agonist (liraglutide) increases serum concentrations of adiponectin and decreases those of resistin, compared to conventional treatment with sulfonylureas (glibenclamide) [105].

(iii) The third relevant point is related to MALFD—T2DM—GLP-1 plasma level interactions and the plasma pro-inflammatory cytokine profile.

The last point of this investigation relates to TNFα, IL-6, IL-1β, and IL-17. We observed that the drop in plasma GLP-1 concentrations was associated with moderate or even excessive production of these four cytokines. Some authors show elevated cytokines, particularly C-reactive protein (CRP), tumor necrosis factor-alpha (TNFα), interleukin-6 (IL-6), and interleukin-1β (IL-1β) in patients with T2DM. Several studies have described that TNFα often acts as an initiator of the inflammatory cascade, while IL-6 amplifies it and links directly to metabolic dysfunction, mainly insulin resistance. These studies suggest that IL-6 levels correlate more robustly with the overall presence and severity of MAFLD, whereas a TNFα increase is particularly notable in the progression to more severe fibrosis and cell death [106].

It is essential to emphasize that IL-6 plays a pivotal role in liver fibrosis, which has diverse etiologies. Indeed, the IL-6 signaling cascade represents a key plasma biomarker for assessing the severity of liver fibrosis. Therapeutically, the use of antibodies targeting the IL-6 signaling pathway represents a novel and potentially promising medical approach for treating liver fibrosis. In MAFLD, IL-6 is primarily expressed in different liver cell populations: hepatocytes, Kupffer cells, and hepatic stellate cells (HSCs). This is explained by the catabolic effects of IL-6 as a pro-inflammatory cytokine with pleiotropic biological activities. In addition to amplifying inflammatory responses, IL-6 also induces lymphocyte differentiation and proliferation, facilitates HSC activation, and contributes to the development of liver fibrosis [107]. IL-6 exerts its effects via HSC activation and hepatic fibrosis by stimulating the JAK/STAT3 (Janus kinase/signal transducer and activator of transcription 3) signaling pathway [108].

Several meta-analyses demonstrate that GLP-1 receptor agonists (GLP-1 RAs) exert significant anti-inflammatory effects, notably by decreasing IL-6 in patients with T2DM [109]. GLP-1 RAs exert immunomodulatory effects on cytokines and chemokines. GLP-1 RAs (such as liraglutide and semaglutide) reduce levels of IL-1β, IL-6, IL-18, and TNFα and increase levels of IL-10. These agents modulate the TGF-β/SMAD (transforming growth factor-β/small mothers against decapentaplegic) and AMPK (AMP-activated protein kinase) signaling pathways to attenuate fibrosis and oxidative damage [110]. Furthermore, clinical trials have shown that short-term infusion of supraphysiological concentrations of GLP-1 in T2DM lead to decreased IL-6 concentrations [111]. However, this does not exclude the possibility that MAFLD + T2DM subjects, as in our study, may insidiously develop NASH. It has recently been described that MAFLD rapidly progresses to NASH without apparent clinical signs and persists asymptomatically in T2DM [112].

It is important to understand the interactions between the cytokines IL-1β and IL-17 and MAFLD in diabetes. IL-1β promotes MAFLD progression by contributing to inflammation, inhibiting fatty acid oxidation, and increasing fat synthesis in hepatocytes. It plays a key role in the evolution from simple steatosis, such as MAFLD or NAFLD, to more severe non-alcoholic steatohepatitis (NASH) and fibrosis, such as cirrhosis. High levels of IL-1β are found in patients with liver steatosis, and blocking IL-1β signaling in animal models reduces liver steatosis and inflammation [113]. IL-1β exerts its pro-inflammatory effects by activating a signaling cascade via IL-1R1, which in turn triggers the activation of cell necrosis transcription factors such as NF-κB, leading to the biosynthesis of pro-inflammatory cytokines [114]. High plasma expression of IL-1β has been observed in patients with MAFLD (group II), associated with the progression of hepatic steatosis to steatohepatitis and hepatic fibrosis. Some studies have demonstrated that peroxisome proliferator-activated receptor gamma (PPARγ) antagonists are therapeutically effective in NASH, while PPARδ activation stimulates mitochondrial beta-oxidation of fatty acids and inhibits hepatic metabolic coupling of liposynthesis and gluconeogenesis.

These data highlight that IL-1β may play a potential therapeutic role via activation of its signaling pathway in response to the tissue damage induced by MAFLD and progression to fibrosis [115]. Interleukin-17A (IL-17) is a cytokine that is characteristic of Th17 helper T cells and is primarily produced by hepatocytes and Kupffer cells (KCs) in the liver. IL-17 exacerbates hepatic steatosis and inflammation, particularly in MAFLD or MASLD, by promoting lipid accumulation and the recruitment of inflammatory cells. It worsens the progression of MAFLD to NASH and cirrhosis, representing a target for potential therapeutic interventions, as our study shows [116]. It is important to emphasize that IL-17 leads to the activation of HSCs, which contributes to liver fibrosis by stimulating the expression of profibrotic factors such as TGF-β. It has been observed that IL-17 acts synergistically with TGF-β, further activating HSCs, thereby inducing elevated collagen production and exacerbating liver fibrosis [117]. These observations have been confirmed by experimental studies that support the deleterious effects of IL-17A in the development of liver fibrosis. The effects of IL-17A are exerted through two main pathways: (1) expression of IL-17A in the hepatic interstitium activates HSCs, thereby promoting elevated collagen synthesis; and (2) stimulation of endothelial cells and fibroblasts by IL-17 induces the secretion of cytokines, chemokines, and cell adhesion factors. This activation inhibits extracellular matrix degradation and promotes fibroblast proliferation. Overall, IL-17-mediated immune responses negatively affect liver tissue, leading to hepatic fibrosis [118]. The effects of GLP-1 on IL-17 metabolism have not been described in the literature; however, as we noted previously with other proinflammatory cytokines, GLP-1 receptor agonists (GLP-1RAs) can promote the expression of multiple anti-inflammatory cytokines in MAFLD [119], including IL-17 [120]. GLP-1 inhibits IL-17 production by stimulating insulin sensitivity, as IL-17 induces an insulin-resistant state [121].

It is crucial to note that increased IL-17 levels and hepatic fibrosis are similar in group IV patients, but not similar in group II (steatosis without diabetes). Several studies argue that this difference is because of the degree of hepatic insulin resistance (HOMA-IR in our study) in patients with MAFLD. The more acute stage of hepatic insulin resistance leads to higher hepatic infiltration of plasma NEFA (non-esterified fatty acids) resulting from the adipose tissue lipolysis (Table 2). NEFA are pro-inflammatory and exert lipotoxic effects [122]. In our study, we found a positive correlation between IL-17 levels and severity of fibrosis (supported by the very high FLI levels) in group IV. Recently, some studies have shown that plasma IL-17 levels are increased even in the absence of liver fibrosis. However, these same studies have shown that pro-fibrotic TGF-β1 (transforming growth factor-beta 1) levels are continuously increasing and positively correlated with hepatic fibrosis. The data indicate that liver fibrosis regulation is complex in MAFLD [123].

(iv) The fourth pertinent point is related to MALFD—T2DM—GLP-1 plasma level interactions and sex-gender disparities.

We observed in our study that T2DM is more common in women compared to men, and that disordered GLP-1 secretion and GLP-1 depletion are more pronounced in women than in men. In this study, the percentage of T2DM women participants is higher than men participants (53% versus 47%, respectively, *p* = 0.001). This may be explained by the higher insulin resistance status in women than in men since adolescence [124]. Several mechanisms are proposed to explain gender-sex differences. Women appear to be more predisposed to T2DM risk factors compared to men, which is linked to the metabolic syndrome, with generally stronger associations in women than men [125], specifically with obesity in women as a prominent T2DM risk factor [126]. In addition, hormonal fluctuations related to reproductive function are specific to women and do not occur in men. Pregnancies may reveal pre-existing metabolic abnormalities, leading to the diagnosis of gestational diabetes, which appears to be the most important risk factor for progression to patent T2DM [127]. In addition, menopause increases the cardiometabolic risk profile in women, but not in men, which explains why women with T2DM have a higher relative cardiovascular risk [128]. These data could partially explain the superiority of women relative to men in the incidence of diabetes disease. In addition, women are more sensitive to treatment with GLP-1 analogs than men [129]. Conversely, MAFLD is more common in men than in women. The prevalence of MAFLD in men increases from early adulthood [130], while in women, rates increase mainly after menopause, affecting liver fibrosis and inflammation differently [131]. According to a relevant study from Pusan National University in South Korea, women may be less at risk of developing MAFLD because they produce FPR2 (formyl peptide protein 2) in greater quantities than men. FPR2 expression was higher in healthy female hepatocytes and livers than in males, and liver damage is exacerbated in female mice with FPR2 deletion. Estrogens, which are female hormones, play a significant role in FPR2 production, and estradiol induces FPR2 expression, thus protecting hepatocytes and the liver from damage. FPR2 modulates inflammatory responses in several organs; however, its role in the liver is unknown [132].

## 4. Patients and Methods

### 4.1. Informed Consent Statement and Ethical Considerations

This clinical study protocol (Algiers Incretin-T2DM-MALFD Study) was approved by the Ethics Committee of the Algerian Ministry of Public Health (ECAMPH) and conformed to the principles outlined in the declaration of Helsinki (http://www.wma.net). Ethical approval code: the permits and ethical rules have been achieved according to the Executive Decree no. 10–90 (10 March 2010) completing the Executive Decree no. 04–82 and no. 379, 381, and 384 (page 5) of Official Gazette of the Algerian republic no. 50 (30 August 2020) of the Algerian Government, establishing the terms and approval modalities. An informed consent form was signed by each participant.

### 4.2. Participants and Clinical Protocol Design

**Figure 7:**
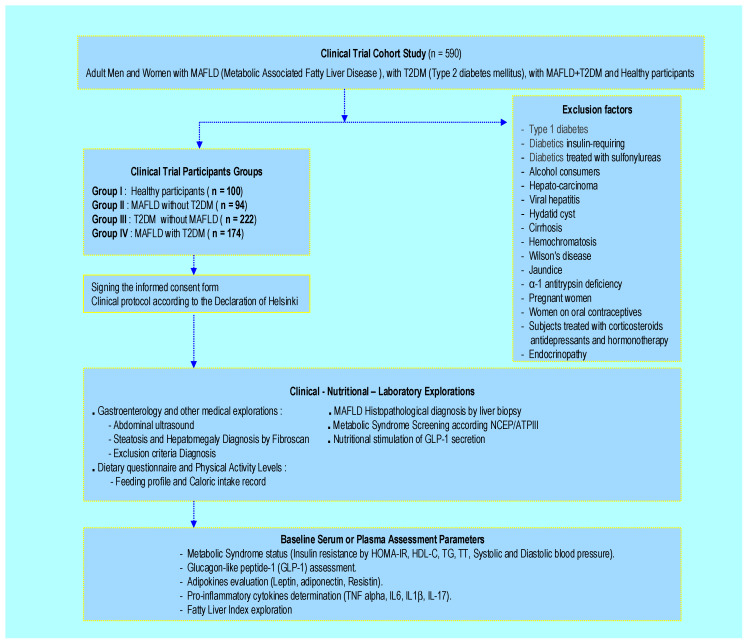
The clinical protocol design for the study involving participants with metabolic dysfunction-associated fatty liver disease (MAFLD-group II), type 2 diabetes mellitus (T2DM-group III), and T2DM associated with MAFLD (group IV) compared to healthy participants (group I). This clinical investigation was a randomized, multicenter, cross-sectional and observational case-control study carried out between November 2022 and December 2024. We included in the study 590 adult participants, aged between 35 and 57 years, including 215 men (M) and 375 women (W). The sample size was estimated using the Cochrane’s formula. All participants were classified according to age and sex, with a sex ratio of men/women = 0.57. Diabetic participants were treated with metformin 300 mg/24 h, without associated a sulfonylurea. Group II was not treated. No participants were insulin-requiring. The drug doses were stable throughout the study. The diabetes duration and the presence of MAFLD in group IV were variable, ranging between 5 and 10 years.

This longitudinal, randomized, multicenter cross-sectional and observational case-control clinical investigation was carried out between November 2022 and December 2024. All participants were admitted to the diabetology unit at Mohamed Seghir Nekkache Hospital and the diabetology-cardiology unit at Bab El Oued University Hospital Center (UHC), Mohamed Lamine Debaghine (MLD) of Algiers, Algeria. All the study parameters measurements were evaluated in the Biochemistry and Genetics Laboratory, UHC-MLD of Algiers. We included in the study 590 adult participants, aged between 35 and 57 years, including 215 men (M) and 375 women (W). The sample size was estimated using the Cochrane formula. This clinical investigation was undertaken as shown in Figure 7.

- 100 healthy participants, non-alcohol consumers and non-smokers (Group I)
- 94 MAFLD participants without T2DM (Group II)
- 222 T2DM participants without MAFLD (Group III)
- 174 MAFLD participants with T2DM comorbidity (Group IV)

Diabetic participants were treated with metformin 300 mg/24 h without a sulfonylurea medication. No participants were insulin-requiring. The drug doses were stable throughout the study. Group IV was not treated, because there is no specific medication for MAFLD. T2DM duration varied between 5 and 10 years.

The study is mainly based on hygiene and dietary measures. The diabetes duration and the presence of MAFLD in Group IV were variable, ranging between 5 and 10 years. In this study, we excluded all subjects with type 1 diabetes, endocrinopathy, alcohol consumers, hepato-carcinoma, viral hepatitis, hydatid cyst cirrhosis, hemochromatosis, Wilson’s disease, jaundice, or α-1 antitrypsin deficiency, diabetics requiring insulin, diabetics treated with sulfonylureas, pregnant women, and subjects taking oral contraceptives, corticosteroids, antidepressants, or hormonotherapy.

### 4.3. Radiological MAFLD Diagnosis

As part of the day hospital program, the MAFLD diagnosis was confirmed by abdominal ultrasound, histological liver biopsy examination, and biochemical profiles of liver function, whether or not associated with hepatomegaly [133]. All study subjects were diagnosed with MAFLD according to the radiological protocol described by Chan et al. [134]. Non-invasive liver fibrosis assessment included fibrosis-4 index (Fib-4 index) and MAFLD fibrosis score. Ultrasound scans were performed after a 12-h fast in a medical imaging center by a single radiologist. A 3.5-MHz transducer (Toosbee, Toshiba, Tokyo, Japan) was attached to a Fibroscan and used to obtain a sagittal view of the right lobe of the liver in relation to the right kidney. This gastroenterological technique allows detection of vibration-controlled transient elastography scores to predict liver-related events in steatotic liver disease [135]. MAFLD severity was assessed using a four-grade test scale: (i) Grade 0: normal echogenicity; (ii) Grade 1: slight diffuse increase in fine echoes in the liver parenchyma with normal visualization of the diaphragm; (iii) Grade 2: moderate to moderate-diffuse increase in echoes with slightly impaired visualization of the intrahepatic vascular system and that of the diaphragm; and (iv) Grade 3: marked increase in fine echoes with poor border of the right posterior lobe or non-visualization of the intrahepatic vascular system and that of the diaphragm. All participants were examined by the same physician.

### 4.4. Fatty Liver Index and MAFLD Diagnosis

As the diagnosis of MAFLD relies on ultrasound, which can be operator-dependent, we suggest calculating the fatty liver index (FLI) retrospectively using available data (BMI, GGT, WC, triglycerides). The fatty liver index (FLI) was developed by Bedogni et al. [136]. This index allows for the early and non-invasive identification of patients at risk of MAFLD, thus facilitating rapid stratification, including referral for specialized gastroenterology ultrasound. In our study, FLI was used for the detection of MAFLD in patients with type 2 diabetes. The FLI encompasses four variables: body mass index (BMI), serum triglyceride levels, waist Circumference (WC), and serum gamma-glutamyl transferase (GGT) levels, according to the following formula: e^x^/(1 + e^x^) × 100, where x = 0.953 × log_e_TG (mmol/L) + 0.139 × BMI (kg/m^2^) + 0.718 × log_e_ GGT (IU/L) + 0.053 × WC (cm) − 15.745. The result is expressed as a score between 0 and 100. FLI was calculated at baseline and categorized into three groups: low (<30), moderate (30–60), and high (>60). A score below 30 excludes the presence of hepatic steatosis.

### 4.5. Metabolic Syndrome (MetS) Screening

MetS was confirmed according to NCEP/ATPIII criteria [137]. MetS was identified by the presence of three or more disorders of MetS clusters, as follows: (1) visceral obesity; (2) high plasma triglyceride level; (3) low plasma HDL-cholesterol level; (4) high fasting plasma glucose; and (5) blood pressure disturbance. Insulin resistance was calculated by the homeostasis model assessment insulin resistance (HOMA-IR) method: HOMA index = fasting glucose (mmol/L) × fasting insulin (mU/L)/22.5 [138]. However, it is important to emphasize that, compared to the euglycemic-hyperinsulinemic clamp reference test, the HOMA-IR score has its limitations. It primarily involves subjects with normal waist circumference and BMI; insulin secretion may be physiologically low (confirmed by decreased C-peptide levels); the conditions under which blood samples are analyzed (fasting < 10 h, pain during vein blood collection) can increase cortisol levels, which has a hyperglycemic effect; potential drug interactions (oral antidiabetic drugs, such as sulfonylureas) can affect the result-; the whole blood storage time which can alter red blood cell hemolysis; and the serum sample storage time can affect insulin assessment. The percentage of body fat (%BF) was calculated using the following formula: (1.2 × BMI) + (0.23 × age) − (10.8 × S) − 5.4 (S is the sex correction factor) [139]. The SBP (systolic blood pressure) and DBP (diastolic blood pressure) were measured in the prone position from both arms, three times and 2 min after 10 min of rest using a validated Omron 705 CP type BP monitor (Omron Healthcare Europe BV, Amsterdam, The Netherlands) [140].

### 4.6. Plasma Samples and Biochemical Analysis

The participants were admitted to the hospital at 7 am after 12 h of fasting before therapeutic treatment. Blood samples were centrifuged at 3000 rpm for 10 min, and plasma was obtained. Fasting plasma samples were immediately put on ice and kept frozen at −80 °C until analyses were performed. Fasting plasma glucose, triglycerides (TGs), total cholesterol (TC), high-density lipoprotein cholesterol (HDL-C), alanine aminotransferase (ALAT), aspartate aminotransferase (ASAT), gamma-glutamyltranspeptidase (GGT), alkaline phosphatase (AP), and total bilirubin (TB) were determined by enzymatic methods using an automatic biochemical analyzer (Cobas Integra 400^®^ analyzer, Roche Diagnostics, Meylan, France). Plasma glycosylated hemoglobin (HbA1C) wase determined by turbidimetry (Roche Diagnostic Systems, Basel, Switzerland). Low-density lipoprotein cholesterol (LDL-C) was calculated using Friedewald’s formula [LDL-C (mg/dL) = TC − HDL-C − TG/5.0]. The criterion for detecting low-grade inflammation has been determined by plasma high-sensitive C-reactive protein (Hs-CRP) level and ferritin, assessed using immuno-turbidimetric methods on a chemical Synchron analyzer LX^®^20 PRO. Plasma non-esterified fatty acids (NEFAs) were determined by microfluorimetry. Fibrinogen was evaluated by the chronometric Von Clauss methods using a ACL TOPTM hemostasis analyzer (Biolabo, Maizy, France). Insulin concentrations were determined by RIA (radioimmunoassay) using commercially available kits (human insulin-specific RIA kit, EMD Millipore Corporation St. Louis, MO, USA). Plasma adipokines (leptin, adiponectin, resistin) were measured using an enzyme-linked immunosorbent assay on a BiotekELX800 human ELISA reader (Marshall scientific, Hampton, NY, USA). ELISA kits were provided by IBL International GmbH (Hamburg, Germany). The assay sensitivities were 0.5 ng/mL for leptin, 0.012 ng/mL for resistin, and 0.185 μg/mL for adiponectin. Plasma pro-inflammatory cytokines (TNF (tumor necrosis factor-alpha), IL-6, IL-1β, and interleukin IL-17) were measured in EDTA tubes by ELISA (Marshall scientific, Hampton, NY, USA), with sensitivities of 2, 1, 1–2, and 0.1 pg/mL, respectively. Standards, reagents, and test samples were prepared and analyzed according to the manufacturer’s instructions.

### 4.7. Plasma Glucagon-like Peptide-1 (GLP-1) Assessment

Plasma GLP-1 was measured in the fasting and postprandial state, when maximal incretin secretion occurs. We opted for a nutritional stimulation that most closely mimics physiological GLP-1 secretion, compared to the oral glucose test. Since GLP-1 secretion is biphasic (two peaks of secretion, as with insulin), it was measured 15 and 60 min after meal digestion. Food intake comprised 820 kcalories composed of carbohydrates, fats, and proteins. This standard test meal consists of two eggs, 250 mL of semi-skimmed milk, two rusks, 50 g of jam, and 250 mL of orange juice. The test is performed after a 12-h fast and 24-h abstinence from medication. Blood samples were collected in EDTA tubes containing a DPP-IV inhibitor at a final concentration of 0.01 mmol/L of buffer (Novo Nordisk A/S, Bagsværd, Denmark). In our study, we opted to measure total GLP-1, containing the most insulinotropic fragments, particularly fragments 7–36, 7–37, and 9–36, with very low levels of fragments 1–36 and 1–37. For calculations, we used an average value of the measurements obtained at 15 and 60 min, expressed as GLP-1. GLP-1 was measured using an ELISA method (with a sensitivity of 0.6 pmol/L) according to the supplier’s instructions (ALPCO Diagnostics, 26-G Keewaydin Dr, Salem, NH 03079, USA, https://www.alpco.com/store/total-glp-1-elisa-7-36-and-9-36.html, accessed on 1 November 2022).

### 4.8. Statistical Analysis

Considering our investigation involved a randomized cohort, all data are measured as normally distributed series. All statistical analyses were performed with Epi-info version 5 and Statview version 5 (Abacus Concepts, Berkeley, CA, USA). Student’s *t*-test and one-way ANOVA were used. Results were expressed as mean ± standard error (SEM), with a significance level of *p* < 0.05. The ANOVA test was used to compare means between the MAFLD, T2DM, and MAFLD + T2D groups versus healthy participants. Pearson’s correlation coefficient (r) was applied to quantify the associations between GLP-1 and metabolic syndrome clusters, adipokines (leptin, adiponectin, resistin), and inflammatory markers (TNF-alpha, IL-6, IL-1β, IL-17). A multivariate linear regression model was used to examine the independent relationship between plasma GLP-1 levels and disease status (MAFLD alone, T2DM alone, or MAFLD -T2DM association), using healthy subjects as the control group after adjustment for BMI, age, sex, and T2DM duration as covariates. We calculated a standardized β, as this coefficient indicates the change in standard deviations of the independent factor or confounding factors (age, BMI, sex-gender, T2DM duration).

## 5. Conclusions and Future Perspectives

This study analyzes a clinical plasma GLP-1 profiles in type 2 diabetes mellitus (T2DM) patients with or without hepatic steatosis (MAFLD). The change in the GLP-1 secretion is correlated with a state of insulin resistance that disrupts liver function through increased lipogenesis and intrahepatocyte lipid infiltration, laying the foundation for MAFLD. Hepatic dysfunction and hyperactivity of visceral adipose tissue alter the adipocytokine profile and impair glycoregulation, which explains the coexistence of T2DM with MAFLD. These interactions maintain insulin resistance and minimize the beneficial action of incretins via the effects of GLP-1. Finally, a longitudinal study would be desirable to confirm the role of GLP-1 as an accurate transition biomarker between non-alcoholic fatty liver disease and glucose intolerance in diabetic subjects. This study may serve as a therapeutic target for GLP-1 in steatotic and diabetic subjects exhibiting acute inflammatory shock and oxidative stress. Although the severity of MAFLD is higher in men compared to women before menopause, the influence of sex on the pathophysiology of MAFLD remains poorly understood. It would be interesting to conduct a longitudinal therapeutic study based on GLP-1 analogs such as liraglutide in MALFD subjects with low plasma GLP-1 levels and to determine if MAFLD does not progress to hepatic fibrosis and with preserved glucose tolerance (diabetes prevention). The hypothesis would be that the GLP-1 analog would inhibit the apoptosis/necrosis process in mitochondrial signaling pathways via its receptors.

## Figures and Tables

**Table 1:** Clinical trial cohort characterization according anthropometric status in MAFLD, T2DM and MAFLD with T2DM Participants groups.

P/G	Group I N = 100	Group II N = 94	Group III N = 222	Group IV N = 174
Sex-gender repartition (%)	43 (M) 57 (W)	54 (M) 46 (W)	96 (M) 126 (W)	75 (M) 99 (W)
Age (year)	38 ± 3 ^(M)^	48 ± 1 ^(M)^	52 ± 3 ^(M)^	57 ± 1 ^(M)^
	35 ± 4 ^(W)^	45 ± 2 ^(W)^	50 ± 1 ^(W)^	49 ± 1 ^(W)^
Δ Age (year)	36 ± 3	46 ± 1	51 ± 1	53 ± 1
Body Weight (Kg)	67 ± 2 ^(M)^	76 ± 4 ^(M)^	75 ± 5 ^(M)^	79 ± 3 ^(M)^
	68 ± 3 ^(W)^	77 ± 3 ^(W)^	79 ± 4 ^(W)^	83 ± 2 ^(W)^
Δ Body Weight (Kg)	67.5 ± 2.5	76.5 ± 3.5	77 ± 4.5	81 ± 2.5
BMI (Kg/m^2^)	21 ± 2 ^(M)^	29 ± 1 ^(M)^	28 ± 3 ^(M)^	29 ± 1 ^(M)^
	22 ± 2 ^(W)^	30 ± 1 ^(W)^**	33 ± 1 ^(W)^**	33 ± 1 ^(W)^***
Δ BMI	21.5 ± 2	29.5 ± 1 **	30.5 ± 2 **	31 ± 1 ***
WC (cm)	85 ± 1 ^(M)^	99 ± 2 ^(M)^***	102 ± 3 ^(M)^***	104 ± 4 ^(M)^***
	77 ± 2 ^(W)^	97 ± 1 ^(W)^***	106 ± 1 ^(W)^***	106 ± 2 ^(W)^***
Δ WC	81 ± 1.5	98 ± 1.5 ***	104 ± 2 ***	105 ± 3 ***
WC/WH ratio	0.86 ± 0.05 ^(M)^	1.05 ± 0.06 ^(M)^***	1.10 ± 0.02 ^(M)^***	1.07 ± 0.04 ^(M)^***
	0.83 ± 0.03 ^(W)^	0.91 ± 0.01 ^(W)^***	0.89 ± 0.07 ^(W)^***	0.93 ± 0.01 ^(W)^***
Δ WC/WH ratio	0.85 ± 0.04	0.98 ± 0.03 ***	0.99 ± 0.04 ***	1.01 ± 0.02 ***
BF (%)	2.22 ± 0.60 ^(M)^	30.7 ± 1.81 ^(M)^***	16.5 ± 1.91 ^(M)^***	32.2 ± 1.65 ^(M)^***
	12.0 ± 0.41 ^(W)^	42.0 ± 1.66 ^(W)^***	18.7 ± 1.25 ^(W)^***	44.1 ± 1.19 ^(W)^***
Δ BF (%)	7.11 ± 0.51	36.3 ± 1.71 ***	17.6 ± 1.52 ***	38.5 ± 1.91 ***

P: parameters; G: group; MAFLD: Metabolic Associated Fatty Liver Disease. T2DM: Type 2 Diabetes Mellitus. Group I: Healthy control participants; Group II: MAFLD participants without T2DM; Group III: T2DM participants without MAFLD; Group IV: MAFLD participants with T2DM comorbidity; M: Men; W: Women; N: total number of participants; BMI: body mass index; WC: waist circumference; WH: waist hips; BF: body fat percentage. Δ: Average values between M and W. The mean values are assigned from the standard error to the mean (X ± SD). The degree of significance is calculated for a risk of error α = 5%. The comparison of means is established both between the groups II, III, and IV versus group I (Healthy group). *** *p* < 0.001, ** *p* < 0.01.

**Table 2:** Clinical trial cohort classification according metabolic syndrome in MAFLD, T2DM and MAFLD with T2DM Participants groups.

P/G	Group I (N = 100)	Group II (N = 94)	Group III (N = 222)	Group IV (N = 174)
GLP-1 (pmole/L)	11.7 ± 1.71 ^(Fs)^	9.50 ± 2.52 ^(Fs)^*	8.80 ± 1.52 ^(Fs)^***	6.62 ± 1.60 ^(Fs)^***
	34.5 ± 3.81 ^(PP)^	20.7 ± 4.18 ^(PP)^*	14.6 ± 2.25 ^(PP)^***	10.9 ± 2.70 ^(PP)^ ***
GLP-1 (pmole/L)	23.2 ± 2.14 ^(M)^	16.3 ± 1.55 ^(M)^ ***	11.7 ± 1.43 ^(M)^ ***	9.51 ± 1.33 ^(M)^ ***
	23.1 ± 1.41 ^(W)^	13.9 ± 1.77 ^(W)^ ***	10.4 ± 1.86 ^(W)^ ***	8.68 ± 1.77 ^(W)^ ***
Δ GLP-1 (pmole/L)	23.1 ± 2.76	15.1 ± 3.35 ***	11.7 ± 1.88 ***	8.76 ± 2.15 ***
Glycemia (mmol/L)	4.67 ± 0.39	6.27 ± 0.09 **	8.71 ± 0.33 ***	9.21 ± 0.42 ***
HbA1_C_ (%)	5.53 ± 0.80	5.62 ± 0.70 ***	6.17 ± 0.18 ***	6.39 ± 0.11 ***
HbA_1c_ (mmol/mol)	37 ± 5.35	38 ± 4.05 ***	44.1 ± 3.80 ***	46.1 ± 1.46 ***
Insulinemia (pmol/L)	63 ± 3.47	165 ± 5.62 ***	299 ± 21 ***	510 ± 38 ***
Homa-IR Index	1.98 ± 0.10	6.65 ± 0.26 ***	17.1 ± 1.54 ***	30.6 ± 2.48 ***
Triglycerides (mmol/L)	0.72 ± 0.04 ^(M)^	1.65 ± 0.15 ^(M)^ ***	1.67 ± 0.23 ^(M)^ ***	2.31 ± 0.33 ^(M)^ ***
	0.71 ± 0.01 ^(W)^	1.33 ± 0.17 ^(W)^ ***	1.80 ± 0.36 ^(W)^ ***	2.18 ± 0.77 ^(W)^ ***
Δ Triglycerides (mmol/L)	0.72 ± 0.04	1.50 ± 0.19 ***	1.76 ± 0.10 ***	2.22 ± 0.14 ***
Total Cholesterol (mmol/L)	3.98 ± 0.22	5.05 ± 0.40 ***	4.89 ± 0.57 ***	5.54 ± 0.93 ***
HDL- Chol (mmol/L)	1.24 ± 0.04 ^(M)^	1.10 ± 0.05 ^(M)^	1.05 ± 0.03 ^(M)^	1.00 ± 0.04 ^(M)^
	1.57 ± 0.08 ^(W)^	1.23 ± 0.05 ^(W)^	1.14 ± 0.06 ^(W)^	1.06 ± 0.02 ^(W)^
LDL- Chol (mmol/L)	2.49 ± 0.02	3.21 ± 0.15	3.49 ± 0.12 ***	4.29 ± 0.06 ***
SBP (mm Hg)	125 ± 15	126 ± 16	129 ± 13	135 ± 17
DBP (mm Hg)	80 ±3 ± 3.81	79.8 ± 5.7	80.7 ± 6.3	81.3 ± 7.4
Total bilirubin (µmol/L)	9.91 ± 0.68	11.4 ± 0.17	11.2 ± 0.68	12.1 ± 0.34
AST (IU/l)	21.7 ± 1.20	30.6 ± 2.61	26.4 ± 1.82	41.0 ± 4.68 ***
ALT (IU/l)	26.8 ± 1.09	76.3 ± 5.90 ***	29.1 ± 1.58	82.1 ± 5.98 ***
AST/ALT Ratio	0.81 ± 0.06	0.66 ± 0.04 ***	0.90 ± 0.05	0.63 ± 0.08 ***
GGT (IU/l)	21.8 ± 2.09 ^(M)^	72.3 ± 4.55 ^(M)^ ***	60.5 ± 3.43 ^(M)^ ***	83.7 ± 4.33 ^(M)^ ***
	21.1 ± 1.11 ^(W)^	66.9 ± 3.77 ^(W)^ ***	52.4 ± 2.86 ^(W)^ ***	80.6 ± 3.77 ^(W)^ ***
Δ GGT (IU/l)	21.9 ± 2.20	70.4 ± 4.42 ***	52.4 ± 3.66 ***	83.7 ± 5.24 ***
AP (UI/L)	79.3 ± 3.37	91.4 ± 5.11	86.5 ± 4.64	98.0 ± 6.92 ***
Iron (g/L)	1.28 ± 0.05	1.33 ± 0.02	1.55 ± 0.07 *	1.95 ± 0.07 *
Ferritin (pmol/L)	147 ± 11	204 ± 3 ***	172 ± 5	309 ± 4 ***
Hs-CRP (mg/L)	3.5 ± 1.2	7.70 ± 0.6 ***	5.61 ± 0.1 **	9.44 ± 0.8 ***
NEFA (µmol/L)	290 ± 36	510 ± 51 **	689 ± 123 ***	897 ± 78 ***
FLI	24.9 ± 2.32 ^(M)^	56.2 ± 8.33 ^(M)^ ***	31.7 ± 3.43 ^(M)^ ***	89.9 ± 5.33 ^(M)^ ***
	6.42 ± 1.23 ^(W)^	43.9 ± 7.44 ^(W)^ ***	25.4 ± 4.86 ^(W)^ ***	78.3 ± 7.77 ^(W)^ ***
Δ FLI	15.5 ± 1.77	50.5 ± 7.88 ***	28.5 ± 4.14 **	84.1 ± 6.55 ***

P: parameters; G: group; MAFLD: Metabolic Associated Fatty Liver Disease. T2DM: Type 2 Diabetes Mellitus. Group I: Healthy control participants; Group II: MAFLD participants without T2DM; Group III: T2DM participants without MAFLD; Group IV: MAFLD participants with T2DM comorbidity; M: Men; W: Women; N: total number of participants; GLP-1: Glucagon-Like Peptide-1. Fs: Fasting state. PP: Post-prandial or fed state. Δ GLP-1: average values at 15 and 60 min. Homa-IR: Homeostasis Model Assessment-Insulinresistance. Chol: Cholesterol. HDL/LDL-C: high-density/low-density lipoprotein cholesterol. SBP: Systolic Blood Pressure. DBP: Diastolic Blood Pressure. AST: Aspartate aminotransferase; ALT: Alanine aminotransferase; GGT: Gamma-glutamyl transferase. AP: Alkaline phosphatase. Hs-CRP: High sensitive C reactive Protein. NEFA: non-esterified fatty acids. FLI: Fatty Liver Index. Δ: Average values between M and W. Mean values are assigned the standard error to the mean (X ± SEM). The degree of significance is calculated for a risk of error α = 5%. The comparison of means is established between the MAFLD, T2DM and MAFLD + T2DM groups versus Healthy group. * *p* < 0.05; ** *p* < 0.01; *** *p* < 0.001.

**Table 3:** Multivariate linear regression model analysis predictor of GLP-1 levels after adjustment for potential confounders Covariates: BMI, Age, Sex-gender repartition and T2DM duration in MAFLD with or without T2DM.

Model	Standardised β	*p*-Value
BMI (kg/m^2^)	−0.647	<0.001
Age (Year)	−0.633	<0.001
Sex-gender	−0.688	<0.001
T2DM duration (Year)	−0.283	<0.001

GLP-1: Glucagon-Like Peptide-1. MAFLD: Metabolic Associated Fatty Liver Disease. T2DM: Type 2 Diabetes Mellitus. BMI: Body Mass iIndex. M: Men; W: Women. *p*-value < 0.05 was considered to be statistically significant.

**Table 4:** Clinical trial cohort distribution according Plasma Adipokines profile in MAFLD, T2DM and Diabetic Steatosis Participants.

P/G	Group I N = 100	Group II N = 94	Group III N = 222	Group IV N = 174
Leptin (ng/mL)	3.25 ± 0.43 ^(M)^	4.84 ± 0.6 ^(M)^*	7.34 ± 1.57 ^(M)^***	8.57 ± 0.99 ^(M)^***
	5.22 ± 0.40 ^(F)^	12.2 ± 2.6 ^(F)^***	21.7 ± 3.18 ^(F)^***	26.6 ± 0.28 ^(F)^**
Δ Leptin (ng/mL)	4.23 ± 0.41	8.52 ± 1.51 ***	14.5 ± 2.51 ***	17.6 ± 0.53 ***
Adiponectin (µg/mL)	6.04 ± 0.12 ^(M)^	3.42 ± 0.24 ^(M)^***	3.54 ± 0.33 ^(M)^***	1.65 ± 0.51 ^(M)^*
	8.07 ± 1.15 ^(F)^	4.97 ± 0.76 ^(F)^**	4.22 ± 0.48 ^(F)^**	3.69 ± 0.36 ^(F)^**
Δ Adiponectin (µg/mL)	7.05 ± 0.63	4.19 ± 0.51 ***	3.88 ± 0.41 ***	2.67 ± 0.43 ***
Resistin (ng/mL)	3.24 ± 0.81 ^(M)^	5.47 ± 0.63 ^(M)^**	6.87 ± 0.73 ^(M)^**	8.33 ± 0.58 ^(M)^**
	3.80 ± 0.62 ^(F)^	4.85 ± 0.37 ^(F)^**	6.10 ± 0.91 ^(F)^**	7.90 ± 0.45 ^(F)^**
Δ Resistin (ng/mL)	3.52 ± 0.71	4.98 ± 0.51 ***	6.48 ± 0.82 ***	8.11 ± 0.51 ***
L/A	(0.53 ± 0.04) × 10^−3 (M)^	(1.41 ± 0.01) × 10^−3 (M)^*	(2.07 ± 0.03) × 10^−3 (M)^***	(6.23 ± 0.01) × 10^−3 (M)^**
	(0.64 ± 0.05) × 10^−3 (F)^	(2.54 ± 0.02) × 10^−3 (F)^*	(5.14 ± 0.04) × 10^−3 (F)^***	(5.67 ± 0.02) × 10^−3 (F)^**
Δ L/A	(0.46 ± 0.04) × 10^−3^	(1.97 ± 0.01) × 10^−3 *^	(3.61 ± 0.03) × 10^−3 *^**	(5.95 ± 0.01) × 10^−3 *^**

P: parameters; G: group; Group I: Healthy control participants; Group II: MAFLD participants without T2DM; Group III: T2DM participants without MAFLD; Group IV: MAFLD participants withT2DM; M: male; F: female; N: total number of participants. L/A: Leptin/Adiponectin ratio. Δ: Average values between M and W. Mean values are assigned the standard error to the mean (X ± SEM). The degree of significance is calculated for a risk of error α = 5%. The comparison of means is established between the MAFLD, T2DM and MAFLD + T2DM groups versus the healthy group. *: *p* < 0.05; **: *p* < 0.01; ***: *p* < 0.001.

**Table 5:** Clinical trial cohort repartition according plasma pro inflammatory cytokines profile in MAFLD, T2DM and Diabetic Steatosis.

P/G	Group I N = 100	Group II N = 94	Group III N = 222	Group IV N = 174
TNF-α (pg/mL)	6.06 ± 0.33	7.77 ± 0.15 **	6.50 ± 0.23	9.92 ± 1.57 ***
IL-6 (pg/mL)	7.62 ± 1.43	11.6 ± 0.23 ***	9.66 ± 0.85 *	19.3 ± 0.56 ***
IL-1β (pg/mL)	3.25 ± 0.45	9.12 ± 0.52	7.69 ± 2.12	11.9 ± 1.49
IL-17 (pg/mL)	34.7 ± 4.51	41.2 ± 10.1 ***	67.4 ± 11.1	92.1 ± 12.9 ***

P: parameters; G: group; Group I: Healthy control participants; Group II: MAFLD participants without T2DM; Group III: T2DM participants without MAFLD; Group IV: MAFLD participants withT2DM; M: male; F: female; N: total number of participants. TNF-α: Tumor necrosis factor-alpha. IL-6: Interleukin-6. IL-1β: Interleukin-1β. IL-17: Interleukin-17. Mean values are assigned the standard error to the mean (X ± SEM). The degree of significance is calculated for a risk of error α = 5%. The comparison of means is established between the MAFLD, T2DM and MAFLD + T2DM groups versus the healthy group. *: *p* < 0.05; **: *p* < 0.01; ***: *p* < 0.001.

## Data Availability

The data presented in this study are available on request from the corresponding author.
